# Trends in popularity of some morphological traits of purebred dogs in Australia

**DOI:** 10.1186/s40575-016-0032-2

**Published:** 2016-04-05

**Authors:** Kendy T. Teng, Paul D. McGreevy, Jenny-Ann L. M. L. Toribio, Navneet K. Dhand

**Affiliations:** Faculty of Veterinary Science, School of Life and Environmental Sciences, The University of Sydney, JD Stewart Building (B01), Camperdown, NSW 2050 Australia; Faculty of Veterinary Science, School of Life and Environmental Sciences, The University of Sydney, R.M.C. Gunn Building (B19), Camperdown, NSW 2050 Australia; Faculty of Veterinary Science, School of Life and Environmental Sciences, The University of Sydney, JL Shute Building (C01A), 425 Werombi Road, Camden, NSW 2570 Australia

**Keywords:** Purebred dogs, Dog popularity, Dog height, Dog size, Cephalic index, Brachycephalic, Disease predisposition, Australia

## Abstract

**Background:**

The morphology of dogs can provide information about their predisposition to some disorders. For example, larger breeds are predisposed to hip dysplasia and many neoplastic diseases. Therefore, longitudinal trends in popularity of dog morphology can reveal potential disease pervasiveness in the future. There have been reports on the popularity of particular breeds and behavioural traits but trends in the morphological traits of preferred breeds have not been studied.

**Methods:**

This study investigated trends in the height, dog size and head shape (cephalic index) of Australian purebred dogs. One hundred eighty-one breeds derived from Australian National Kennel Council (ANKC) registration statistics from 1986 to 2013 were analysed. Weighted regression analyses were conducted to examine trends in the traits by using them as outcome variables, with year as the explanatory variable and numbers of registered dogs as weights. Linear regression investigated dog height and cephalic index (skull width/skull length), and multinomial logistic regression studied dog size.

**Results:**

The total number of ANKC registration had decreased gradually from 95,792 in 1986 to 66,902 in 2013. Both weighted minimal height (*p =* 0.014) and weighted maximal height (*p <* 0.001) decreased significantly over time, and the weighted cephalic index increased significantly (*p <* 0.001). The odds of registration of medium and small breeds increased by 5.3 % and 4.2 %, respectively, relative to large breeds (*p <* 0.001) and by 12.1 % and 11.0 %, respectively, relative to giant breeds (*p <* 0.001) for each 5-year block of time.

**Conclusions:**

Compared to taller and larger breeds, shorter and smaller breeds have become relatively popular over time. Mean cephalic index has increased, which indicates that Australians have gradually favoured breeds with shorter and wider heads (brachycephalic). These significant trends indicate that the dog morphological traits reported here may potentially influence how people select companion dogs in Australia and provide valuable predictive information on the pervasiveness of diseases in dogs.

**Electronic supplementary material:**

The online version of this article (doi:10.1186/s40575-016-0032-2) contains supplementary material, which is available to authorized users.

## Plain English Summary

Some diseases in dogs are related to certain physical characteristics. For example, larger breeds have a higher risk of getting hip dysplasia and certain neoplastic diseases while breeds with wider and shorter heads, such as Pug and French bulldog, are more likely to experience breathing problems and dystocia. Therefore, if we know the trends in popularity of dogs of a certain morphology, we may be able to predict disease pervasiveness.

The study aimed to investigate the trends in the height, dog size and head shape of Australian purebred dogs. The numbers of dogs registered within the 181 breeds in Australian National Kennel Council (ANKC) every year from 1986 to 2013 were obtained and analysed.

The total number of ANKC registration had decreased from 95,792 in 1986 to 66,902 in 2013. Compared to taller and larger breeds, shorter and smaller breeds have become relatively popular over time. Also, the data suggest that Australians increasingly favour dogs with shorter and wider heads for whose welfare veterinarians often express concern [1, 2].

The results indicate that dog height, dog size and dog head shape may potentially influence how people select companion dogs in Australia and provide valuable predictive information on trends in disease prevalence, enabling the veterinary profession and industry to prepare for potential future caseloads.

## Background

Breed predispositions to disease are well recognized [3]. Regardless of breed, the morphologies of dogs often imply an individual’s predisposition to some disorders [4]. For example, hip dysplasia [5, 6], gastric dilatation volvulus [7, 8] and many neoplastic diseases [9] frequently occur in larger sized breeds of dogs. In contrast, smaller breeds of are predisposed to myxomatous mitral valve disease [10] and tracheal collapse [11–13]. Additionally, brachycephalic breeds are susceptible to several health concerns such as brachycephalic airway obstruction syndrome (BAOS) [14, 15], dystocia in dams [16], digestive disorders and multiple eye conditions [4].

People prefer different types of dogs and use various criteria for selecting their household dogs, which can be influenced by human lifestyle, cultural backgrounds, media exposure, education, etc. Therefore, changes in any of these may impact the decision making process in dog selection. For example, the purpose of keeping dogs has been shifting from specific functionalities to primarily companion in the western world and this would potentially have had impacts on people’s choice of dogs [17]. Several studies have been conducted to understand the trends in the popularity of dogs and their reasons. A previous study on trends in the popularity of purebred dogs suggested that breeds became highly favoured mainly by chance without a specific trend [18]. It was also proposed that social influence (fashion) is the primary influence on the popularity of companion dog breeds, which is related to media exposure [19, 20], and that people showed no preference to breeds with sound health and good behaviours [19, 21]. However, although the appearance and the size of the dog are often considered while selecting a companion dog, we are not aware of any research conducted to reveal the potential trends in the popularity of morphological traits of dogs.

This study was conducted to investigate potential trends in some morphological traits of Australian purebred dogs, including dog height, dog size and cephalic index (skull width divided by skull length), by analysing a 28-year-long (1986–2013) Australian National Kennel Council (ANKC) dog registration dataset (Additional file 1) [22].

## Methods

### Data collection and management

ANKC has recorded and published the registration numbers of each ANKC-recognised breed on their website each year since 1986 [22]. For every year from 1986 to 2013, the numbers of dogs registered for 181 breeds were drawn from the ANKC registration report. For some breeds, there were more than one observation due to the breeds’ variation, such as Chihuahua, whose hair can be long or smooth; therefore, 204 observations (each breed and its variations) were independently included in the study.

Height and weight references of breeds were obtained from the ANKC studbook [23] or from the Encyclopedia of Dog Breeds (EoDB) [24], if not available in the studbook. Height is the distance from a dog’s withers to the ground. The lowest and highest height values in one breed were chosen as the minimal and maximal heights for the breed, respectively, and the minimal and maximal weight were determined likewise. For example, as the minimal and maximal heights in male Saint Bernard are 70 and 90 cm and 65 and 80 cm for female, respectively, 65 and 90 cm were then considered as the minimal and maximal height values for Saint Bernard. The weight range for each breed was used to assign breeds to one of the four size groups, namely, small (less than 10 kg), medium (10 kg to less than 25 kg), large (25 to less than 40 kg) and giant (40 kg and over) based on the widely applied criteria [25–27]. A breed belonged to a size group when its whole weight range located in the group; otherwise, it would be classified into the group whose range covered the average of minimal and maximal weight of the breed. Breeds with only one weight value were categorised according to the specified value. Cephalic index and standard deviations of both sexes for 80 breeds were drawn from the literature and calculated for each breed [28]. Larger cephalic index indicates the head shape of the breed is more brachycephalic, whereas breeds with smaller values have a more dolichocephalic shape of head. Characteristics and their source of the most popular 20 breeds in Australia in 2013 are presented in Table 1 and Additional file 2 lists the information of all ANKC-recognised breeds.

**Table 1:** The morphologies of the most popular 20 Australian National Kennel Council (ANKC)recognised breeds in 2013

Breed	Source of Height	Height Range (cm)	Source of Weight	Weight Range (kg)	Dog size	Cephalic Index (±SD^a^ )
American Staffordshire Terrier	ANKC	(43, 48)	EoDB	(25.9, 30.4)	Large	67.40 (±3.34)
Australian Cattle Dog	ANKC	(43, 51)	EoDB	(15.9, 20.4)	Medium	61.60 (±8.75)
Border Collie	ANKC	(46, 53)	EoDB	(13.6, 20.4)	Medium	56.70 (±4.32)
Boxer	ANKC	(53, 61)	EoDB	(22.7, 36.3)	Large	66.75 (±6.01)
British Bulldog	EoDB	(30, 38)	EoDB	(18.1, 22.7)	Medium	86.60 (±4.34)
Bull Terrier	EoDB	(53, 56)	EoDB	(22.7, 31.8)	Large	55.60 (±10.90)
Cavalier King Charles Spaniel	EoDB	(30, 33)	ANKC	(5.4, 8.2)	Small	76.25 (±4.78)
Cocker Spaniel	ANKC	(38, 41)	ANKC	(13.0, 14.5)	Medium	48.85 (±4.12)
French Bulldog	EoDB	(28, 33)	EoDB	(NA^b^, 12.7)	Medium	101.55 (±2.42)
German Shepherd Dog	ANKC	(55, 65)	ANKC	(22.0, 40.0)	Large	50.40 (±8.62)
Golden Retriever	ANKC	(51, 61)	EoDB	(25.0, 34.0)	Large	56.05 (±3.54)
Great Dane	EoDB	(79, 89)	ANKC	(46.0, 54.0)	Giant	56.60 (±4.56)
Jack Russell Terrier	ANKC	(25, 30)	ANKC	(5.0, 6.0)	Small	61.45 (±2.75)
Labrador Retriever	ANKC	(55, 57)	EoDB	(25.0, 36.3)	Large	55.95 (±4.80)
Poodle (Toy)	EoDB	(NA^b^, 25)	EoDB	(1.8, 3.6)	Small	NA^b^
Pug	EoDB	(25, 28)	ANKC	(6.3, 8.1)	Small	98.55 (±6.74)
Rhodesian Ridgeback	ANKC	(61, 69)	EoDB	(31.8, 38.6)	Large	50.45 (±3.10)
Rottweiler	ANKC	(56, 68)	EoDB	(36.3, 61.2)	Giant	63.55 (±2.95)
Schnauzer (Miniature)	EoDB	(30, 36)	EoDB	(5.9, 6.8)	Small	53.40 (±2.44)
Staffordshire Bull Terrier	ANKC	(36, 41)	ANKC	(11.0, 17.0)	Medium	76.15 (±6.32)

Legend: The morphologies of the 20 most popular dog breeds in Australian National Kennel Council (ANKC) registry in 2013, based on data sourced from the ANKC breed standards and the Encyclopedia of Dog Breeds (EoDB). Cephalic Index data were required from a peer-reviewed paper [28]. Characteristics of all ANKC-recognised breeds are presented in Additional file 2

^a^SD: standard deviation; ^b^NA: missing data

### Statistical methods

Data were extracted into and cleaned in Microsoft Excel 2010 spreadsheets. Descriptive statistics were generated for the overall trends in the number of registrations and in each morphological trait in Microsoft Excel 2010. The means of minimal height, maximal height, minimal weight, maximal weight and cephalic index, weighted by the registration numbers, over the 28 years were calculated and plotted, as well as the proportions of registration number of each size group. Correlation between minimal height, maximal height, minimal weight and maximal weight were calculated. To insure that the contribution of each observation is proportional to the number of registration, weighted regression analyses were then conducted to examine trends in each trait, using the trait as the outcome variable, year as the explanatory variable and numbers of registered dogs as weights to account for different numbers of registration of each breed each year. Linear regression investigated dog height and cephalic index and multinomial logistic regression studied dog size by using the SAS statistical program, 9.3th edition (SAS Institute, Cary, NC. USA). Normality and homoscedasticity were assessed by visual inspection of residual and residual-versus-fitted plots. A two sided P-value < 0.05 was considered statistically significant.

## Results

### Descriptive statistics

**Fig. 1:**
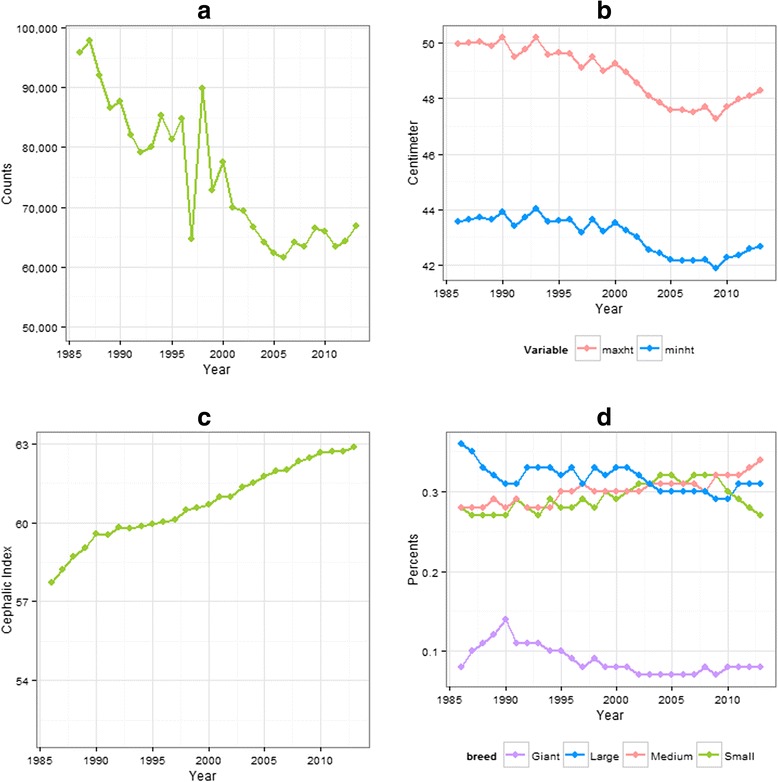
The descriptive statistics results of all variables each year from 1986 to 2013. The **a** total registration number, **b** weighted means of minimal and maximal heights, **c** weighted mean of cephalic index, and **d** proportions of each dog size group

The total number of ANKC registration had decreased gradually from 95,792 in 1986 to 66,902 in 2013. A precipitous fluctuation in numbers registered between 1997–1998 was noted (Fig. 1a). The numbers of breeds and their variations which have at least one registry increased from 144 to 183 from 1986 to 2013. While the majority of the height records were sourced from the ANKC studbook (*n =* 140) and the remaining from the EoDB (*n =* 64), similar numbers of weight records were extracted from the ANKC studbook (*n =* 86) and the EoDB (*n =* 94). Standard Poodle had no maximal height value, whereas Smooth and Wire Fox Terrier, Toy Poodle, Shih Tzu, Tibetan Spaniel and Welsh Terrier had no minimal height values. In addition, 23 breeds and both variations of German Spitz (German Spitz Klein and German Spitz Mittel) were not classified into any size groups due to no weight records being available from the sources consulted. In total, there were 54 small breeds, 62 medium breeds, 42 large breeds, and 22 giant breeds for which data were used in this study.

**Fig. 2:**
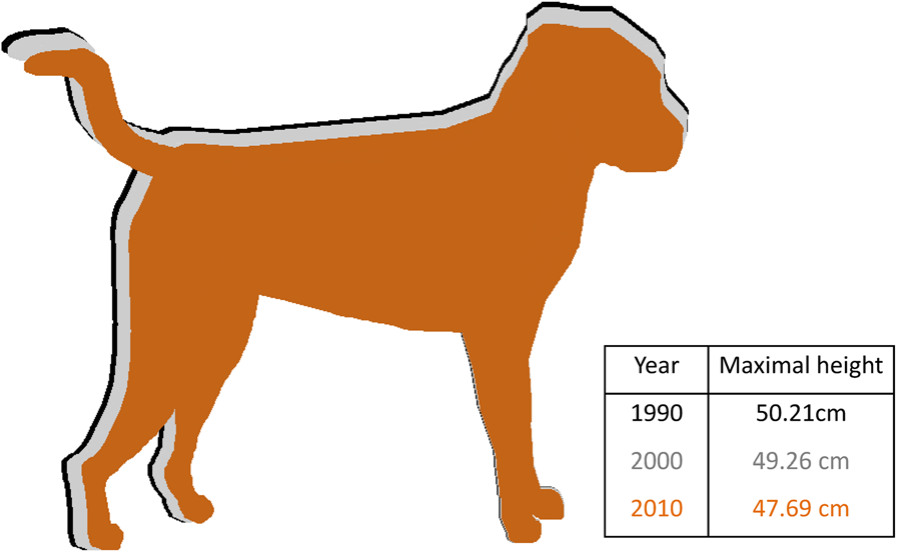
The change of the mean dog maximal height in Australia every 10-year block of time

The weighted mean of maximal height decreased from 50.0 cm in 1986 to 48.3 cm in 2013, and weighted mean of minimal height decreased from 43.6 cm in 1986 to 42.7 cm in 2013 (Figs. 1b and 2). Weighted mean of cephalic index increased from 57.7 in 1986 to 62.9 in 2013 (Fig. 1c). The proportions of small, medium and large breeds ranged between 0.26 to 0.36 in the 28-year period, while the proportion of giant breed only ranged from 0.07 to 0.14 (Fig. 1d). The proportion of medium sized breeds steadily increased over time.

### Modelling results

Correlations between minimal and maximal heights, and minimal and maximal weights were 0.98 (*p <* 0.001) and 0.96 (*p <* 0.001), respectively. Correlations between minimal height and weight, and maximal height and weight were 0.82 (*p <* 0.001) and 0.86 (*p <* 0.001), respectively.

The results suggested that both weighted minimal height and maximal height decreased significantly over time while the weighted cephalic index increased significantly during the period (Table 2). The multinomial logistic model results indicated that odds of registration of medium and small breeds increased by 5.3 % and 4.2 %, respectively, relative to large breeds (*p <* 0.001) and by 12.1 % and 11.0 %, respectively, compared to giant breeds (*p <* 0.001) (Table 3) for each 5-year block of time.

**Table 2:** Weighted linear regression model results for the trends in dog height and cephalic index of Australian National Kennel Council (ANKC) recognised breed

Outcome variable	Parameter	b	S.E.	95 % CI^a^	t-value	*P*- value
Maximal Height	Intercept	50.62	0.48	(49.68, 51.56)	105.60	<0.001
	Year	−0.10	0.03	(−0.16, −0.04)	−3.34	<0.001
Minimal Height	Intercept	44.04	0.43	(43.20, 44.89)	102.33	<0.001
	Year	−0.07	0.03	(−0.12, −0.01)	−2.46	0.014
Cephalic Index	Intercept	58.18	0.47	(57.26, 59.11)	123.63	<0.001
	Year	0.17	0.03	(0.16, 0.23)	5.83	<0.001

Legend: Weighted linear regression model results with the maximal height, minimal height and cephalic index of the breeds (*n =* 204) in Australian National Kennel Council (ANKC) as outcome variables, year (1986 – 2013) as the predictor and numbers of registered dogs each breed each year in ANKC as weights over the 28 years

^a^95 % CI: 95 % confidence interval

**Table 3:** Weighted multinominal regression model results for the trend in dog size of Australian National Kennel Council (ANKC) recognised breedsᅟ

Outcome variable	Category	Intercept	b	S.E.	*P* -value	OR	95 % CI^b^
Dog size	Giant	-	0	-	-	1	-
	Small	0.894	0.104	0.002	<.001	1.110	(1.106 1.113)
	Medium	0.894	0.114	0.002	<.001	1.121	(1.117, 1.125)
	Large	1.010	0.063	0.002	<.001	1.065	(1.062, 1.068)

Legend: Weighted multinominal regression model results with the dog size as outcome variable, year (1986 – 2013) as the predictor and numbers of registered dogs each breed each year in Australian National Kennel Council registry as weights over the 28 years

^a^OR: odds ratio; ^b^95 % CI: 95 % confidence interval

## Discussion

This study reveals previously undocumented trends in morphological traits of purebred dogs in Australia by using the ANKC registry dataset. Shorter and smaller breeds, as well as breeds with larger cephalic index, show growing popularity in ANKC registrations in the 28 years. These significant trends provide valuable predictive information on the pervasiveness of diseases in Australian companion dogs.

The total registration numbers have been continuously declining with a difference of about 30,000 over the 28 years. This can be due to decreases purely in numbers registered to ANKC, purebred dog numbers in Australia, dog numbers in Australia, or any combination of these factors. It is noted that the numbers of ANKC memberships have decreased from 54,590 in 1995 to 33,119 in 2013 [29]. However, new dog registrations would mostly depend on the number of newborn pedigree dogs rather than the number of ANKC members, and a breeder can have more than one breeding bitch. The profile of pedigree dog breeders may have been compromised by media focus on inherited disorders and puppy farms; this in combination with the promotion of adoptions may have reduced the demand for purebred dogs in Australia. In addition, the recent surges of popularity of designer breeds, those are crossbred with two different breeds of dogs, is noticed by veterinarians. The trend can also partially result from reduced dog population size in Australia. Pet industry figures indicate a decreasing trend from 1998 to 2009 [30], although there are also recent predictions of an upturn in Australian dog numbers [31].

Changes to human lifestyle, to dwelling types and to the roles of dogs in human societies can affect dog owners’ decisions about dog acquisition including preferred breed or type. From 1995 to 2010, the proportion of single separate house purchases (excluding semi-detached/row and townhouse/terrace house) decreased and flat/unit/apartment purchases increased among first home buyers with a mortgage in Australia [32], which may indicate that space available for dogs would also have shrunk. Moreover, the major purposes of dog ownership nowadays have changed from certain functionalities such as hunting and guarding properties, for which dogs are more likely to be larger, to purely companionship, a purpose which can be fulfilled by dogs of various size [17]. These may be two of the many possible explanations of trends in dog height and dog size observed in the current study.

The results show that breeds with a larger cephalic index have steadily become more popular, which indicates that Australians have gradually favoured dogs with shorter and wider heads (brachycephalic) more than those with longer and thinner heads (dolichocephalic). The brachycephaly boom seems to be worldwide. In agreement with our results, brachycephalic breeds such as English Bulldogs, French Bulldogs, Boxers and Pugs, have been becoming increasingly popular in the United Kingdom (UK) over recent years [33], and the numbers of Bulldogs and French bulldogs registered with the American Kennel Club have increased by 69 % and 476 %, respectively, in the past decade [34]. The typical skull shape of a breed often aligns with the breed’s original purpose. For example, medium and large size brachycephalic breeds have stronger bite force [35], which seems to align with their common historic role in baiting and fighting context [36]. On the other hand, a dolichocephalic morphology is associated with a breed’s ability as a visual hunter [37]. However, as functionality has become a minor incentive to acquire dogs, the popularity of breeds with larger cephalic index may have two possible causes instead of functionality. Firstly, the neotenic appearance of brachycephalic dogs may account for the popularity [38]. Many research studies have shown that the infantile facial features stimulate affective and caretaking behavioural responses in human adults, which has the evolutionary benefits of increasing the survival of the vulnerable individuals [39–42]. These cute features, defined by Konrad Lorenz (1943), are called “baby schema” [43], including large head, round face, chubby cheeks, high and protruding forehead, big eyes, small nose and mouth, etc. Interestingly, baby schema effect has been observed not merely in human infant but also cross species [44–46]. The head of brachycephalic dogs is characterised by a round and short face, open orbitae, a small and short nose, which accord with the baby schema features [38]. Therefore, baby schema effect may explain the increasing popularity of brachycephalic breeds. Secondly, a flux in perceived aesthetics may be responsible for the phenomenon. It has been confirmed that human behaviours and preferences can be contagious without rationale [47]. One study endorses this theory by demonstrating that fads play a major factor in choosing the breed of companion dogs [19].

Accompanying the trends in the prevalence of the morphological traits revealed in this study, we predict corresponding changes in the patterns of disease occurrence in dogs in Australia: diseases among smaller breeds and brachycephalic breeds are expected to be seen increasingly by the veterinary profession in Australia. The predicted increase in veterinary observation of diseases that predominantly affect these types of dogs can be tracked by examining and analysing electronic patient health records from primary care veterinary clinics longitudinally.

With the increase of smaller and brachycephalic dogs, conditions leading to mortality in small breeds (urogenital diseases, degenerative diseases, metabolic diseases) will potentially be seen more, compared to those that have an increased risk of death in larger breeds, such as diseases of musculoskeletal and gastrointestinal systems and many neoplastic diseases [48]. Through reviewing the literature, we have identified 13 common diseases in smaller breeds, compared to 25 diseases in larger breeds listed in the Additional file 3. In larger breeds, 28 % of the diseases are musculoskeletal, 20 % are nervous/sensory, 16 % are cardiovascular, and 36 % belong to the rest organ systems. In contrast, in smaller breeds, no clusters of diseases of specific organ systems have been noticed. Among the dog-size predisposed diseases, patellar luxation (PL), portosystemic shunt (PSS) and mammary tumour (MT) show different forms of predispositions in smaller and larger breeds. While PL was originally recognised as a condition to which smaller breeds were predisposed [49], the occurrence in larger breeds appears to be increasing [50, 51]. Medial PLs are the predominant condition regardless of dog size [52], whereas the lateral form is reported more frequently in larger breeds [51, 53]. PSS is generally more prevalent in smaller breeds [54–56], especially in the form of extrahepatic PSSs [54, 57]. In contrast, intrahepatic PSS cases are seen more commonly in larger breeds [54, 58, 59]. Although MT is more frequently seen in smaller breeds of dogs [60], the MTs with greater malignancy [61] and thus a more profound effect on life expectancy are encountered among larger breeds [60]. We would like to acknowledge that the common diseases and disease occurrence may not be the same in different continents/countries due to the divergent gene pool although many of the disease predispositions are commonly recognised worldwide.

Concerns for the welfare of brachycephalic dogs have been highlighted recently and, as reported by our results and the literature, this issue is likely to become an increasing concern for veterinarians and dog owners in Australia and worldwide. In New Zealand, 4 of the top 5 dog breeds considered by veterinarians to be unsuitable for continued breeding due to compromised health and welfare are brachycephalic breeds [1]. The life expectancy is estimated 4 years lower in highly brachycephalic breeds than those not (8.6 years vs 12.7 years) [62]. BAOS, resulting in mild to life-threatening respiratory dysfunction [14], has received attention in the UK following the growing popularity of brachycephalic breeds in that country [33, 63]. However, a UK study showed that approximately half of the owners of BAOS affected dogs seem unaware of BAOS in their dogs [64], which indicates that they did not make informed decisions when they purchased them, that dogs might not receive necessarily medical treatment when BAOS emerges, and that the use of affected dogs might persist in breeding programmes. Cephalopelvic disproportion between whelps and dams is thought to be responsible for dystocia in brachycephalic dogs and to lead to inevitable caesarean section [65]. One paper reports caesarean section being performed to deliver over 80 % of litters for registered pedigree bitches of Boston Terrier, English Bulldog and French Bulldog in the UK [66], and the biggest Swedish insurance company applies special rules for reimbursement associated with caesarean section to these three breeds [67]. Additionally, there is some suggestion that pregnant brachycephalic dams often receive caesarean section before natural parturition begins [66]. Other brachycephalic-predisposed conditions include mast cell tumours [68], chemoreceptor system neoplasms [69–71], hydrocephalus [72] and multiple digestive, ocular and dermatological disorders [4, 73].

This is the first trend study in the popularity of canine morphological traits other than those determined solely by breed. By fitting model of random genetic drift, Herzog suggests that the randomness of fashion largely explains the popularity of dog breeds [18, 19]. Another study shows no correlation between popularity and the longevity or the desirable behavioural tendencies of breeds [21]. As our results show linear relationships between certain morphological traits of dogs and time, this may suggest that the preference for morphological features of dogs may be embedded in social changes and trends, such as urbanisation and pursuit of cuteness, which influence people’s criteria for selecting household dogs. By knowing that dog size is often a consideration while choosing a dog along with our results [74], it is reasonable to conclude that dog height and dog size may potentially be one of the major considerations in decision making process of selecting companion dogs in Australia. However, we would also like to acknowledge that there may be other unmeasured factors influencing trends in dog numbers over time that we cannot capture from the data we have.

This study has a number of possible limitations that we wish to acknowledge. Firstly, although the ANKC dog registration dataset has high data integrity with few missing data, the results are highly representative of ANKC-registered dogs, and so may not truly represent either the purebred population or general dog population in Australia. The ANKC estimates that 16.5 % of newborn puppies in Australia in 2014 were from ANKC [75]. That said, since the ANKC is the leading kennel club in Australia, it is plausible that the composition of dog breeds in the Australian purebred population reflects those registered with ANKC. However, this would be based on the assumption that purebred dogs outside of ANCK also strictly follow the ANKC breed standards, which may not be true. Secondly, not all the height and weight records are representative of Australian purebred dogs since some are derived from EoDB, where most standards are from the American Kennel Club. Additionally, both of these sources adopt various standardising methods for different breeds, which limits the consistency of our data. For instance, while most breeds have minimal and maximal height restriction for both sexes, some have a height range for the breed or only a mean height. Thirdly, even though dog size groups are commonly used for research and in everyday veterinary practice, no universal dog sizing criteria can be found from the literature. For the current study, we classified dog size according to dog weight, as is standard practice in academic research and is considered a better predictor of lifespan than dog height [76]. Lastly, although significant linear trends appear in the changes of the morphological traits over time, we could only postulate about the best explanations for the identified trends but were not be able to test the causality in the current study.

## Conclusions

This study identifies that, over the 28-year period (1986–2013), shorter and smaller breeds became relatively popular, compared to taller and larger breeds, and the mean cephalic index increased, suggesting that Australians are tending to prefer breeds with a wider and shorter head. These significant trends indicate that the dog morphological traits reported here may potentially influence how people select companion dogs in Australia and suggest valuable predictive information on the pervasiveness of diseases, enabling the veterinary profession and industry to prepare for potential future caseloads. It would be interesting to examine similar datasets from other countries.

## Additional files

Additional file 1: The registration numbers of all Australian National Kennel Council (ANKC) recognised breeds in ANKC registry from 1986 to 2013. (XLSX 42 kb) Additional file 2: The morphologies of all Australian National Kennel Council (ANKC) recognised breeds, based on data sourced from the ANKC breed standards and the Encyclopedia of Dog Breeds (EoDB). Cephalic Index data were derived from a peer-reviewed paper [28]. Characteristics of the most popular 20 breeds in 2013 are presented in Table 1. (DOCX 44 kb) Additional file 3: Table S1. Disease predispositions in larger breeds of dogs. Table S2 Disease predispositions in smaller breeds of dogs. (DOCX 91 kb)

## Abbreviations

BAOS: Brachycephalic Airway Obstruction Syndrome
ANKC: Australian National Kennel Council
EoDB: Encyclopedia of Dog Breeds
UK: United Kingdom
PL: Patellar Luxation
PSS: Portosystemic Shunts
MT: Mammary Tumours
