# Supplementary material for: Trends in popularity of some morphological traits of purebred dogs in Australia
Source: Canine Genet Epidemiol. 2016 Apr 5;3:2. doi: 10.1186/s40575-016-0032-2 (PMC4820977; doi:10.1186/s40575-016-0032-2)
Supplement: Additional file 3: Table S1. — Disease predispositions in larger breeds of dogs. Table S2 Disease predispositions in smaller breeds of dogs. (DOCX 91 kb) [file 40575_2016_32_MOESM3_ESM.docx]

**Supplementary Table 2 Disease predispositions in larger breeds of dogs**.

| **Organ system** | **Disease** | **Reference** |
| --- | --- | --- |
| Cardiovascular | Dilated cardiomyopathy | [[77](#_ENREF_1), [78](#_ENREF_2)] |
| Cardiovascular | Intrahepatic portosystemic shunts | [54, [58, 5](#_ENREF_3)9] |
| Cardiovascular | Pericardial effusion | [[79](#_ENREF_6), [80](#_ENREF_7)] |
| Cardiovascular | Tricuspid valve dysplasia | [[8](#_ENREF_8)1, [82](#_ENREF_9)] |
| Endocrine | Insulinoma | [83, 84] |
| Gastrointestinal | Gastric dilatation-volvulus | [7, 8] |
| Gastrointestinal | Oral fibrosarcoma | [85] |
| Gastrointestinal | Undifferentiated malignancy | [85] |
| Integument | Hygroma | [86] |
| Musculoskeletal | Cranial cruciate ligament disease | [87] |
| Musculoskeletal | Elbow dysplasia | [88, [8](#_ENREF_18)9] |
| Musculoskeletal | Hip dysplasia | [5, 6, [87](#_ENREF_16)] |
| Musculoskeletal | Masticatory muscle myositis | [90, 91] |
| Musculoskeletal | Osteochondritis dissecans | [[92-](#_ENREF_23)94] |
| Musculoskeletal | Osteosarcomas | [9, 95] |
| Musculoskeletal | Patellar luxation | [[50-](#_ENREF_28)52] |
| Nervous_sensory | Cervical spondylomyelopathy (Wobbler Syndrome) | [96, 97] |
| Nervous_sensory | Degenerative lumbosacral stenosis | [[98-](#_ENREF_33)100] |
| Nervous_sensory | Degenerative myelopathy | [101] |
| Nervous_sensory | Fibrocartilaginous embolism | [[26, 102](#_ENREF_37), 103] |
| Nervous_sensory | Primary intracranial neoplasms | [104] |
| Respiratory | Canine nasosinal tumours | [[105-](#_ENREF_41)107] |
| Respiratory | Laryngeal paralysis | [108-110] |
| Urogenital | Acquired urinary sphincter mechanism incompetence | [111-113] |
| Urogenital | Higher incidence of malignancy in mammary tumours | [60, 114] |

**Supplementary Table 3 Disease predispositions in smaller breeds of dogs.**

| **Organ system** | **Disease** | **Reference** |
| --- | --- | --- |
| Cardiovascular | Chordae tendineae rupture | [115] |
| Cardiovascular | Myxomatous mitral valve disease | [10, 116] |
| Cardiovascular | Portosystemic shunt | [[55](#_ENREF_55), [56](#_ENREF_56), 79] |
|  | Extrahepatic portosystemic shunt | [57, [79](#_ENREF_5)] |
| Gastrointestinal | Oral melanoma | [61, 117] |
| Musculoskeletal | Atlantoaxial subluxation | [[118-](#_ENREF_60)120] |
| Musculoskeletal | Legg-Calve-Perthes disease | [121, 122] |
| Musculoskeletal | Patellar luxation | [49, 52] |
| Nervous_sensory | Caudal occipital malformation syndrome | [72] |
| Nervous_sensory | Congenital hydrocephalus | [72] |
| Nervous_sensory | Necrotizing meningoencephalitis | [72] |
| Respiratory | Tracheal collapse | [[11-](#_ENREF_67)13] |
| Urogenital | Mammary tumours | [[60](#_ENREF_50)] |
| Urogenital | Urolithiasis with calcium oxalate uroliths | [[123-](#_ENREF_70)125] |

**References**

77. Tidholm A, Jönsson L: **A retrospective study of canine dilated cardiomyopathy (189 cases)**. *J Am Anim Hosp Assoc* 1997, **33**(6):544-550.

78. Martin MWS, Stafford Johnson MJ, Celona B: **Canine dilated cardiomyopathy: a retrospective study of signalment, presentation and clinical findings in 369 cases**. *J Small Anim Pract* 2009, **50**(1):23-29.

79. Johnson MS, Martin M, Binns S, Day M: **A retrospective study of clinical findings, treatment and outcome in 143 dogs with pericardial effusion**. *J Small Anim Pract* 2004, **45**(11):546-552.

80. MacDonald KA, Cagney O, Magne ML: **Echocardiographic and clinicopathologic characterization of pericardial effusion in dogs: 107 cases (1985–2006)**. *J Am Vet Med Assoc* 2009, **235**(12):1456-1461.

81. Tidholm A: **Retrospective study of congenital heart defects in 151 dogs**. *J Small Anim Pract* 1997, **38**(3):94-98.

82. Arai S, Griffiths LG, Mama K, Hackett TB, Monnet E, Boon JA, Carter L, Orton EC: **Bioprosthesis valve replacement in dogs with congenital tricuspid valve dysplasia: Technique and outcome**. *J Vet Cardiol* 2011, **13**(2):91-99.

83. Goutal CM, Brugmann BL, Ryan KA: **Insulinoma in Dogs: A Review**. *J Am Anim Hosp Assoc* 2012, **48**(3):151-163.

84. Lunn KF, Page RL: **25 - Tumors of the Endocrine System**. In: Withrow SJ, Vail DM, Page RL, editors. Withrow and MacEwen's Small Animal Clinical Oncology (Fifth Edition). Saint Louis: W.B. Saunders; 2013: 504-531.

85. Liptak JM, Withrow SJ: **22 - Cancer of the Gastrointestinal Tract**. In: Withrow SJ, Vail DM, Page RL, editors. Withrow and MacEwen's Small Animal Clinical Oncology (Fifth Edition). Saint Louis: W.B. Saunders; 2013: 381-431.

86. Pavletic M, Brum D: **Successful closed suction drain management of a canine elbow hygroma**. *J Small Anim Pract* 2015, **56**(7):476-479.

87. Witsberger TH, Villamil JA, Schultz LG, Hahn AW, Cook JL: **Prevalence of and risk factors for hip dysplasia and cranial cruciate ligament deficiency in dogs**. *J Am Vet Med Assoc* 2008, **232**(12):1818-1824.

88. Narojek T, Fiszdon K, Hanysz E: **Canine elbow dysplasia in different breeds**. *Bull Vet Inst Pulawy* 2008, **52**:169-173.

89. Sturaro E, Ojala M, Maki K, Bittante G, Carnier P, Pedrani G, Gallo L: **Results from an explorative screening program for elbow dysplasia in some breeds of dogs in Italy**. *Ital J Anim Sci* 2005, **4**(3):233.

90. Clooten JK, Woods J, Smith-Maxie LL: **Myasthenia gravis and masticatory muscle myositis in a dog**. *Can Vet J* 2003, **44**(6):480.

91. Melmed C, Shelton GD, Bergman R, Barton C: **Masticatory muscle myositis: pathogenesis, diagnosis, and treatment**. *Compendium on Continuing Education for the Practising Veterinarian-North American Edition* 2004, **26**(8):590-605.

92. Denny HR, Gibbs C: **Osteochondritis dissecans of the canine stifle joint**. *J Small Anim Pract* 1980, **21**(6):317-322.

93. Johnston SA: **Osteochondritis Dissecans of the Humeral Head**. *Vet Clin North Am Small Anim Pract* 1998, **28**(1):33-49.

94. Person MW: **Arthroscopic treatment of osteochondritis dissecans in the canine shoulder**. *Vet Surg* 1989, **18**(3):175-189.

95. Ru G, Terracini B, Glickman LT: **Host related risk factors for canine osteosarcoma**. *Vet J* 1998, **156**(1):31-39.

96. da Costa RC: **Cervical Spondylomyelopathy (Wobbler Syndrome) in Dogs**. *Vet Clin North Am Small Anim Pract* 2010, **40**(5):881-913.

97. Lewis DG: **Cervical spondylomyelopathy (‘wobbler’ syndrome) in the dog: A study based on 224 cases**. *J Small Anim Pract* 1989, **30**(12):657-665.

98. Danielsson F, Sjostrom L: **Surgical treatment of degenerative lumbosacral stenosis in dogs**. *Vet Surg* 1999, **28**(2):91-98.

99. Watt PR: **Degenerative lumbosacral stenosis in 18 dogs**. *J Small Anim Pract* 1991, **32**(3):125-134.

100. Worth AJ, Thompson DJ, Hartman AC: **Degenerative lumbosacral stenosis in working dogs: current concepts and review**. *N Z Vet J* 2009, **57**(6):319-330.

101. Siso S, Hanzlicek D, Fluehmann G, Kathmann I, Tomek A, Papa V, Vandevelde M: **Neurodegenerative diseases in domestic animals: a comparative review**. *Vet J* 2006, **171**(1):20-38.

102. De Risio L, Platt SR: **Fibrocartilaginous Embolic Myelopathy in Small Animals**. *Vet Clin North Am Small Anim Pract* 2010, **40**(5):859-869.

103. Neer TM: **Fibrocartilaginous emboli**. *Vet Clin North Am Small Anim Pract* 1992, **22**(4):1017-1026.

104. Song RB, Vite CH, Bradley CW, Cross JR: **Postmortem evaluation of 435 cases of intracranial neoplasia in dogs and relationship of neoplasm with breed, age, and body weight**. *J Vet Intern Med* 2013, **27**(5):1143-1152.

105. Patnaik A: **Canine sinonasal neoplasms: clinicopathological study of 285 cases**. *J Am Anim Hosp Assoc* 1989, **25**(1).

106. Avner A, Dobson JM, Sales JI, Herrtage ME: **Retrospective review of 50 canine nasal tumours evaluated by low-field magnetic resonance imaging**. *J Small Anim Pract* 2008, **49**(5):233-243.

107. Rassnick KM, Goldkamp CE, Erb HN, Scrivani PV, Njaa BL, Gieger TL, Turek MM, McNiel EA, Proulx DR, Chun R *et al*: **Evaluation of factors associated with survival in dogs with untreated nasal carcinomas: 139 cases (1993–2003)**. *J Am Vet Med Assoc* 2006, **229**(3):401-406.

108. Gaber CE, Amis TC, LeCouteur RA: **Laryngeal paralysis in dogs: a review of 23 cases**. *J Am Vet Med Assoc* 1985, **186**(4):377-380.

109. MacPhail CM, Monnet E: **Outcome of and postoperative complications in dogs undergoing surgical treatment of laryng eal paralysis: 140 cases (1985–1998)**. *J Am Vet Med Assoc* 2001, **218**(12):1949-1956.

110. White RAS: **Unilateral arytenoid lateralisation: An assessment of technique and long term results in 62 dogs with laryngeal paralysis**. *J Small Anim Pract* 1989, **30**(10):543-549.

111. Holt PE, Thrusfield MV: **Association in bitches between breed, size, neutering and docking, and acquired urinary-incontinence due to incompetence of the urethral sphincter mechanism**. *Vet Rec* 1993, **133**(8):177-180.

112. Arnold S, Arnold P, Hubler M, Casal M, Rüsch P: **Urinary incontinence in spayed female dogs: frequency and breed disposition**. *Schweiz Arch Tierheilkd* 1988, **131**(5):259-263.

113. de Bleser B, Brodbelt DC, Gregory NG, Martinez TA: **The association between acquired urinary sphincter mechanism incompetence in bitches and early spaying: A case-control study**. *Vet J* 2011, **187**(1):42-47.

114. Itoh T, Uchida K, Ishikawa K, Kushima K, Kushima E, Tamada H, Moritake T, Nakao H, Shii H: **Clinicopathological survey of 101 canine mammary gland tumors: differences between small-breed dogs and others**. *J Vet Med Sci* 2005, **67**(3):345-347.

115. Serres F, Chetboul V, Tissier R, Sampedrano CC, Gouni V, Nicolle AP, Pouchelon J-L: **Chordae tendineae rupture in dogs with degenerative mitral valve disease: prevalence, survival, and prognostic factors (114 cases, 2001–2006)**. *J Vet Intern Med* 2007, **21**(2):258-264.

116. Buchanan JW: **Chronic valvular disease (endocardiosis) in dogs**. *Adv Vet Sci Comp Med* 1977, **21**:75.

117. Ramos-Vara J, Beissenherz M, Miller M, Johnson G, Pace L, Fard A, Kottler S: **Retrospective study of 338 canine oral melanomas with clinical, histologic, and immunohistochemical review of 129 cases**. *Vet Pathol* 2000, **37**(6):597-608.

118. Geary JG, Oliver JE, Hoerlein BF: **Atlanto axial subluxation in the canine**. *J Small Anim Pract* 1967, **8**(10):577-582.

119. Beaver DP, Ellison GW, Lewis DD, Goring RL, Kubilis PS, Barchard C: **Risk factors affecting the outcome of surgery for atlantoaxial subluxation in dogs: 46 cases (1978–1998)**. *J Am Vet Med Assoc* 2000, **216**(7):1104-1109.

120. Thomas WB, Sorjonen DC, Simpson ST: **Surgical management of atlantoaxial subluxation in 23 dogs**. *Vet Surg* 1991, **20**(6):409-412.

121. Piek CJ, Hazewinkel HAW, Wolvekamp WTC, Nap RC, Mey BP: **Long term follow-up of avascular necrosis of the femoral head in the dog**. *J Small Anim Pract* 1996, **37**(1):12-18.

122. LaFond E, Breur GJ, Austin CC: **Breed susceptibility for developmental orthopedic diseases in dogs**. *J Am Anim Hosp Assoc* 2002, **38**(5):467-477.

123. Okafor CC, Lefebvre SL, Pearl DL, Yang M, Wang M, Blois SL, Lund EM, Dewey CE: **Risk factors associated with calcium oxalate urolithiasis in dogs evaluated at general care veterinary hospitals in the United States**. *Prev Vet Med* 2014, **115**(3–4):217-228.

124. Lekcharoensuk C, Lulich JP, Osborne CA, Pusoonthornthum R, Allen TA, Koehler LA, Urlich LK, Carpenter KA, Swanson LL: **Patient and environmental factors associated with calcium oxalate urolithiasis in dogs**. *J Am Vet Med Assoc* 2000, **217**(4):515-519.

125. Lulich JP, Osborne CA, Thumchai R, Lekcharoensuk C, Ulrich LK, Koehler LA, Bird KA, Swanson LL, Nakagawa Y: **Epidemiology of canine calcium oxalate uroliths: identifying risk factors**. *Vet Clin North Am Small Anim Pract* 1999, **29**(1):113-122.
