# Supplementary material for: Trends in popularity of some morphological traits of purebred dogs in Australia
Source: Canine Genet Epidemiol. 2016 Apr 5;3:2. doi: 10.1186/s40575-016-0032-2 (PMC4820977; doi:10.1186/s40575-016-0032-2)
Supplement: Additional file 2: — The morphologies of all Australian National Kennel Council (ANKC) recognised breeds, based on data sourced from the ANKC breed standards and the Encyclopedia of Dog Breeds (EoDB). Cephalic Index data were derived from a peer-reviewed paper [28]. Characteristics of the most popular 20 breeds in 2013 are presented in Table 1. (DOCX 44 kb) [file 40575_2016_32_MOESM2_ESM.docx]

**Supplementary Table 1.** The morphologies of all Australian National Kennel Council (ANKC) recognised breeds, based on data sourced from the ANKC breed standards [1] and the Encyclopedia of Dog Breeds (EoDB) [2]. Cephalic Index data were sourced from a peer-reviewed paper [3]. Characteristics of the most popular 20 breeds in 2013 are presented in Table 1.

| **Breed** | **Source of Height** | **Height Range** | **Source of Weight** | **Weight Range** | **Dog size** | **Cephalix Index ( ±SD )** |
| --- | --- | --- | --- | --- | --- | --- |
| Affenpinscher | ANKC | ( 24, 28 ) | EoDB | ( 3.2, 4.1 ) | Small |  |
| Afghan Hounds | ANKC | ( 55, 74 ) | EoDB | ( 22.7, 27.2 ) | Medium | 49.45 ( ±3.20 ) |
| Airedale Terrier | ANKC | ( 56, 61 ) | EoDB | ( 25.0, 25.0 ) | Medium |  |
| Akita | ANKC | ( 61, 71 ) | EoDB | ( 29.5, 52.2 ) | Giant | 57.35 ( ±6.38 ) |
| Akita (Japanese) | EoDB | ( 58, 71 ) |  |  |  |  |
| Alaskan Malamute | EoDB | ( 58, 64 ) | EoDB | ( 34.0, 38.6 ) | Large | 58.10 ( ±6.75 ) |
| American Staffordshire Terrier | ANKC | ( 43, 48 ) | EoDB | ( 25.9, 30.4 ) | Large | 67.40 ( ±3.34 ) |
| Anatolian Shepherd Dog | ANKC | ( 71, 81 ) | ANKC | ( 40.0, 65.0 ) | Giant |  |
| Australian Cattle Dog | ANKC | ( 43, 51 ) | EoDB | ( 15.9, 20.4 ) | Medium | 61.60 ( ±8.75 ) |
| Australian Kelpie | ANKC | ( 43, 51 ) |  |  |  | 56.75 ( ±6.01 ) |
| Australian Shepherd | ANKC | ( 46, 59 ) | EoDB | ( 18.1, 29.5 ) | Medium | 53.40 ( ±6.51 ) |
| Australian Silky Terrier | ANKC | ( 23, 26 ) | EoDB | ( NA, 3.2 ) | Small |  |
| Australian Stumpy Tail Cattle Dog | ANKC | ( 43, 51 ) |  |  |  |  |
| Australian Terrier | EoDB | ( 25, 28 ) | EoDB | ( 5.4, 6.4 ) | Small | 57.85 ( ±3.36 ) |
| Azawakh | ANKC | ( 60, 74 ) | ANKC | ( 15.0, 25.0 ) | Medium |  |
| Basenji | EoDB | ( 41, 43 ) | EoDB | ( 10.0, 10.9 ) | Medium | 55.80 ( ±6.65 ) |
| Basset Fauve de Bretagne | ANKC | ( 32, 38 ) |  |  |  |  |
| Basset Hound | ANKC | ( 33, 38 ) | EoDB | ( 18.1, 27.2 ) | Medium | 50.05 ( ±6.41 ) |
| Beagle | ANKC | ( 33, 40 ) | EoDB | ( 8.2, 13.6 ) | Medium | 59.75 ( ±2.25 ) |
| Bearded Collie | ANKC | ( 51, 56 ) | EoDB | ( 20.4, 25.0 ) | Medium | 50.55 ( ±8.56 ) |
| Bedlington Terrier | EoDB | ( 39, 42 ) | ANKC | ( 8.0, 10.0 ) | Small |  |
| Belgian Shepherd (Groen) | ANKC | ( 56, 66 ) | ANKC | ( 20.0, 30.0 ) | Large |  |
| Belgian Shepherd (Laek) | ANKC | ( 56, 66 ) | ANKC | ( 20.0, 30.0 ) | Large |  |
| Belgian Shepherd (Malin) | ANKC | ( 56, 66 ) | ANKC | ( 20.0, 30.0 ) | Large |  |
| Belgian Shepherd (Tervn.) | ANKC | ( 56, 66 ) | ANKC | ( 20.0, 30.0 ) | Large |  |
| Bergamasco Shepherd Dog | ANKC | ( 54, 62 ) | ANKC | ( 26.0, 38.0 ) | Large |  |
| Bernese Mountain Dog | ANKC | ( 58, 70 ) | EoDB | ( 31.8, 54.4 ) | Giant | 60.95 ( ±5.13 ) |
| Bichon Frise | EoDB | ( 24, 29 ) | EoDB | ( 4.5, 7.3 ) | Small | 63.55 ( ±8.27 ) |
| Bloodhound | ANKC | ( 58, 69 ) | ANKC | ( 45.5, 50.0 ) | Giant |  |
| Bluetick Coonhound | ANKC | ( 59, 76 ) | ANKC | ( 20.4, 45.5 ) | Large |  |
| Border Collie | ANKC | ( 46, 53 ) | EoDB | ( 13.6, 20.4 ) | Medium | 56.70 ( ±4.32 ) |
| Border Terrier | EoDB | ( 25, 28 ) | ANKC | ( 5.0, 7.0 ) | Small |  |
| Borzoi | ANKC | ( 68, 74 ) | EoDB | ( 27.2, 47.6 ) | Large | 38.65 ( ±8.26 ) |
| Boston Terrier | EoDB | ( 38, 43 ) | EoDB | ( 4.5, 11.3 ) | Small | 89.10 ( ±4.70 ) |
| Bouvier des Flandres | ANKC | ( 59, 68 ) | ANKC | ( 27.0, 40.0 ) | Large |  |
| Boxer | ANKC | ( 53, 61 ) | EoDB | ( 22.7, 36.3 ) | Large | 66.75 ( ±6.01 ) |
| Bracco Italiano | ANKC | ( 55, 67 ) | ANKC | ( 25.0, 40.0 ) | Large |  |
| Briard | ANKC | ( 58, 69 ) | EoDB | ( 22.7, 45.4 ) | Large |  |
| British Bulldog | EoDB | ( 30, 38 ) | EoDB | ( 18.1, 22.7 ) | Medium | 86.60 ( ±4.34 ) |
| Brittany | ANKC | ( 47, 51 ) | EoDB | ( 13.6, 18.1 ) | Medium |  |
| Bull Terrier | EoDB | ( 53, 56 ) | EoDB | ( 22.7, 31.8 ) | Large | 55.60 ( ±10.90 ) |
| Bull Terrier (Miniature) | EoDB | ( 25, 36 ) | EoDB | ( 11.3, 15.0 ) | Medium |  |
| Bullmastiff | ANKC | ( 61, 69 ) | ANKC | ( 41.0, 59.0 ) | Giant | 79.10 ( ±3.22 ) |
| Cairn Terrier | ANKC | ( 28, 31 ) | ANKC | ( 6.0, 7.5 ) | Small | 63.40 ( ±6.18 ) |
| Canaan Dog | ANKC | ( 50, 60 ) | ANKC | ( 18.0, 25.0 ) | Medium |  |
| Canadian Eskimo Dog | ANKC | ( 50, 70 ) | ANKC | ( 18.0, 40.0 ) | Large |  |
| Cane Corso | ANKC | ( 60, 68 ) | ANKC | ( 40.0, 50.0 ) | Giant |  |
| Cavalier King Charles Spaniel | EoDB | ( 30, 33 ) | ANKC | ( 5.4, 8.2 ) | Small | 76.25 ( ±4.78 ) |
| Central Asian Shepherd Dog | ANKC | ( 65, 70 ) | ANKC | ( 40.0, 50.0 ) | Giant |  |
| Cesky Terrier | ANKC | ( 25, 32 ) | ANKC | ( 6.0, 10.0 ) | Small |  |
| Chesapeake Bay Retriever | ANKC | ( 53, 66 ) | ANKC | ( 25.0, 36.3 ) | Large |  |
| Chihuahua (Long) | EoDB | ( 15, 23 ) | EoDB | ( NA, 2.7 ) | Small | 71.85 ( ±3.96 ) |
| Chihuahua (Smooth) | EoDB | ( 15, 23 ) | ANKC | ( 1.8, 2.7 ) | Small | 71.85 ( ±3.96 ) |
| Chinese Crested Dog | ANKC | ( 23, 33 ) | ANKC | ( NA, 5.4 ) | Small | 59.90 ( ±5.31 ) |
| Chow Chow | EoDB | ( 43, 51 ) | EoDB | ( 20.4, 31.8 ) | Large |  |
| Clumber Spaniel | EoDB | ( 43, 51 ) | ANKC | ( 25.0, 34.0 ) | Large |  |
| Cocker Spaniel | ANKC | ( 38, 41 ) | ANKC | ( 13.0, 14.5 ) | Medium | 48.85 ( ±4.12 ) |
| Cocker Spaniel (American) | ANKC | ( 34, 39 ) | EoDB | ( 10.9, 12.7 ) | Medium | 59.40 ( ±6.40 ) |
| Collie (Rough) | ANKC | ( 51, 61 ) | ANKC | ( 18.0, 29.5 ) | Medium | 46.65 ( ±9.55 ) |
| Collie (Smooth) | ANKC | ( 51, 61 ) | ANKC | ( 18.0, 29.5 ) | Medium |  |
| Curly Coated Retriever | EoDB | ( 58, 69 ) | EoDB | ( 27.2, 31.8 ) | Large |  |
| Dachshund (Long) | EoDB | ( 20, 23 ) | ANKC | ( 9.0, 12.0 ) | Medium |  |
| Dachshund (Min. Long) | EoDB | ( 13, 15 ) | ANKC | ( 4.5, 5.0 ) | Small | 46.65 ( ±5.47 ) |
| Dachshund (Min. Smooth) | EoDB | ( 13, 15 ) | ANKC | ( 4.5, 5.0 ) | Small | 46.65 ( ±5.47 ) |
| Dachshund (Min. Wire) | EoDB | ( 13, 15 ) | ANKC | ( 4.5, 5.0 ) | Small | 46.65 ( ±5.47 ) |
| Dachshund (Smooth) | EoDB | ( 20, 23 ) | ANKC | ( 9.0, 12.0 ) | Medium |  |
| Dachshund (Wire) | EoDB | ( 20, 23 ) | ANKC | ( 9.0, 12.0 ) | Medium |  |
| Dalmatian | ANKC | ( 56, 61 ) | EoDB | ( 18.1, 27.2 ) | Medium | 51.55 ( ±4.65 ) |
| Dandie Dinmont Terrier | ANKC | ( 20, 28 ) | EoDB | ( 8.2, 10.9 ) | Small |  |
| Deerhound | EoDB | ( 71, 81 ) | EoDB | ( 34.0, 49.9 ) | Giant |  |
| Dobermann | EoDB | ( 61, 71 ) | EoDB | ( 29.5, 40.8 ) | Large | 46.30 ( ±10.50 ) |
| Dogue de Bordeaux | ANKC | ( 58, 68 ) | ANKC | ( 45.0, 50.0 ) | Giant |  |
| Dutch Shepherd | ANKC | ( 55, 62 ) |  |  |  |  |
| English Setter | ANKC | ( 61, 69 ) | EoDB | ( 22.7, 29.5 ) | Large | 43.55 ( ±4.11 ) |
| English Springer Spaniel | EoDB | ( 46, 53 ) | EoDB | ( 18.1, 22.7 ) | Medium | 50.65 ( ±2.77 ) |
| English Toy Terrier (Black & Tan) | ANKC | ( 25, 30 ) | ANKC | ( 2.7, 3.6 ) | Small |  |
| Eurasier | ANKC | ( 48, 60 ) | ANKC | ( 18.0, 32.0 ) | Large |  |
| Field Spaniel | EoDB | ( 41, 48 ) | ANKC | ( 18.0, 25.0 ) | Medium |  |
| Finnish Lapphund | ANKC | ( 41, 52 ) |  |  |  | 57.35 ( ±8.32 ) |
| Finnish Spitz | ANKC | ( 39, 50 ) | ANKC | ( 7.0, 13.0 ) | Medium |  |
| Flat Coat Retriever | ANKC | ( 56, 61 ) | ANKC | ( 25.0, 36.0 ) | Large |  |
| Fox Terrier (Smooth) | ANKC | ( NA, 39 ) | ANKC | ( 7.0, 8.0 ) | Small | 45.00 ( ±5.66 ) |
| Fox Terrier (Wire) | ANKC | ( NA, 39 ) | ANKC | ( 7.0, 8.0 ) | Small |  |
| Foxhound | ANKC | ( 58, 64 ) | EoDB | ( 25.0, 34.0 ) | Large |  |
| French Bulldog | EoDB | ( 28, 33 ) | EoDB | ( NA, 12.7 ) | Medium | 101.55 ( ±2.42 ) |
| German Hunting Terrier | ANKC | ( 33, 40 ) | ANKC | ( 7.5, 10.0 ) | Small |  |
| German Pinscher | ANKC | ( 43, 48 ) | EoDB | ( 11.3, 15.9 ) | Medium | 50.65 ( ±7.10 ) |
| German Shepherd Dog | ANKC | ( 55, 65 ) | ANKC | ( 22.0, 40.0 ) | Large | 50.40 ( ±8.62 ) |
| German Shepherd Dog (LSC) | ANKC | ( 58, 63 ) | ANKC | ( 22.0, 40.0 ) | Large |  |
| German Shorthaired Pointer | ANKC | ( 58, 66 ) | EoDB | ( 20.4, 27.2 ) | Medium | 49.50 ( ±3.62 ) |
| German Spitz (Klein) | ANKC | ( 23, 29 ) |  |  |  |  |
| German Spitz (Mittel) | ANKC | ( 30, 38 ) |  |  |  |  |
| German Wirehaired Pointer | ANKC | ( 57, 68 ) | EoDB | ( 20.4, 34.0 ) | Large |  |
| Glen of Imaal Terrier | ANKC | ( 35, 36 ) | EoDB | ( 14.9, 15.9 ) | Medium |  |
| Golden Retriever | ANKC | ( 51, 61 ) | EoDB | ( 25.0, 34.0 ) | Large | 56.05 ( ±3.54 ) |
| Gordon Setter | EoDB | ( 58, 69 ) | EoDB | ( 20.4, 36.3 ) | Large | 49.60 ( ±7.83 ) |
| Grand Basset Griffon Vendeen | ANKC | ( 39, 44 ) |  |  |  |  |
| Great Dane | EoDB | ( 79, 89 ) | ANKC | ( 46.0, 54.0 ) | Giant | 56.60 ( ±4.56 ) |
| Greyhound | ANKC | ( 69, 76 ) | EoDB | ( 27.2, 31.8 ) | Large | 46.30 ( ±5.75 ) |
| Griffon Bruxellois | EoDB | ( 23, 28 ) | ANKC | ( 3.6, 4.5 ) | Small | 96.50 ( ±2.31 ) |
| Hamiltonstovare | ANKC | ( 49, 61 ) |  |  |  |  |
| Harrier | ANKC | ( 48, 53 ) | EoDB | ( 15.9, 27.2 ) | Medium |  |
| Havanese | ANKC | ( 23, 27 ) | EoDB | ( 3.2, 5.9 ) | Small | 69.05 ( ±5.81 ) |
| Hungarian Vizsla | ANKC | ( 54, 64 ) | EoDB | ( 20.4, 29.5 ) | Medium | 49.80 ( ±6.78 ) |
| Hungarian Wirehaired Vizsla | ANKC | ( 54, 64 ) | EoDB | ( 20.4, 29.5 ) | Medium |  |
| Ibizan Hound | ANKC | ( 56, 74 ) | EoDB | ( 18.1, 22.7 ) | Medium |  |
| Irish Red & White Setter | EoDB | ( 57, 66 ) |  |  |  |  |
| Irish Setter | EoDB | ( 64, 69 ) | EoDB | ( 27.2, 31.8 ) | Large | 43.60 ( ±3.46 ) |
| Irish Terrier | EoDB | ( 46, 46 ) | EoDB | ( 11.3, 12.3 ) | Medium |  |
| Irish Water Spaniel | ANKC | ( 51, 58 ) | EoDB | ( 20.4, 30.8 ) | Large |  |
| Irish Wolfhound | ANKC | ( 81, 86 ) | ANKC | ( 40.5, 54.5 ) | Giant |  |
| Italian Greyhound | ANKC | ( 32, 38 ) | ANKC | ( 3.6, 4.5 ) | Small | 54.35 ( ±2.90 ) |
| Italian Spinone | ANKC | ( 58, 69 ) | ANKC | ( 29.0, 39.0 ) | Large |  |
| Jack Russell Terrier | ANKC | ( 25, 30 ) | ANKC | ( 5.0, 6.0 ) | Small | 61.45 ( ±2.75 ) |
| Japanese Chin | EoDB | ( 20, 28 ) | ANKC | ( 1.8, 3.2 ) | Small |  |
| Japanese Spitz | ANKC | ( 30, 37 ) |  |  |  | 70.95 ( ±3.61 ) |
| Keeshond | EoDB | ( 41, 48 ) | EoDB | ( 15.9, 20.4 ) | Medium | 58.35 ( ±5.64 ) |
| Kerry Blue Terrier | EoDB | ( 44, 50 ) | ANKC | ( 15.0, 17.0 ) | Medium |  |
| King Charles Spaniel | EoDB | ( 25, 28 ) | ANKC | ( 3.6, 6.3 ) | Small |  |
| Komondor | ANKC | ( 65, 70 ) | ANKC | ( 40.0, 60.0 ) | Giant |  |
| Kuvasz | ANKC | ( 66, 76 ) | ANKC | ( 37.0, 62.0 ) | Giant |  |
| Labrador Retriever | ANKC | ( 55, 57 ) | EoDB | ( 25.0, 36.3 ) | Large | 55.95 ( ±4.80 ) |
| Lagotto Romagnolo | ANKC | ( 41, 48 ) | ANKC | ( 11.0, 16.0 ) | Medium | 58.55 ( ±1.77 ) |
| Lakeland Terrier | EoDB | ( 34, 37 ) | EoDB | ( 7.7, 7.7 ) | Small |  |
| Large Munsterlander | ANKC | ( 58, 65 ) |  |  |  |  |
| Leonberger | ANKC | ( 65, 80 ) |  |  |  | 53.80 ( ±3.69 ) |
| Llasa Apso | EoDB | ( 3, 28 ) | EoDB | ( 5.9, 6.8 ) | Small |  |
| Lowchen | ANKC | ( 25, 33 ) | EoDB | ( 3.6, 8.2 ) | Small |  |
| Maltese | EoDB | ( 23, 25 ) | EoDB | ( 1.8, 3.2 ) | Small | 67.05 ( ±5.15 ) |
| Manchester Terrier | EoDB | ( 38, 41 ) | EoDB | ( 5.4, 10.0+E19 ) | Small |  |
| Maremma Sheepdog | ANKC | ( 60, 73 ) | ANKC | ( 30.0, 45.0 ) | Large |  |
| Mastiff | EoDB | ( 70, 76 ) | EoDB | ( 79.4, 86.2 ) | Giant | 70.75 ( ±5.15 ) |
| Miniature Pinscher | ANKC | ( 25, 30 ) | EoDB | ( 3.6, 4.5 ) | Small |  |
| Neapolitan Mastiff | ANKC | ( 60, 75 ) | ANKC | ( 50.0, 70.0 ) | Giant | 72.60 ( ±4.18 ) |
| Newfoundland | EoDB | ( 66, 71 ) | ANKC | ( 50.0, 69.0 ) | Giant |  |
| Norfolk Terrier | EoDB | ( 23, 25 ) | EoDB | ( 5.0, 5.4 ) | Small |  |
| Norwegian Buhund | EoDB | ( 42, 46 ) |  |  |  |  |
| Norwegian Elkhound | EoDB | ( 41, 47 ) | EoDB | ( 21.8, 25 ) | Medium | 55.15 ( ±2.82 ) |
| Norwich Terrier | EoDB | ( 25, 25 ) | EoDB | ( 5.4, 5.4 ) | Small |  |
| Nova Scotia Duck Tolling Ret. | ANKC | ( 45, 51 ) | ANKC | ( 17.0, 23.0 ) | Medium |  |
| Old English Sheepdog | EoDB | ( 53, 56 ) | EoDB | ( 27.2, 40.8 ) | Large | 58.30 ( ±7.60 ) |
| Otterhound | EoDB | ( 61, 69 ) | ANKC | ( 36.3, 52.2 ) | Giant |  |
| Papillon | ANKC | ( 20, 28 ) | EoDB | ( 1.8, 4.1 ) | Small | 70.65 ( ±3.28 ) |
| Parson Russell Terrier | EoDB | ( 30, 36 ) |  |  |  |  |
| Pekingese | EoDB | ( 15, 23 ) | EoDB | ( NA, 6.4 ) | Small |  |
| Petit Basset Griffon Vendeen | ANKC | ( 34, 38 ) | EoDB | ( 11.3, 15.9 ) | Medium |  |
| Pharaoh Hound | ANKC | ( 54, 64 ) | EoDB | ( 20.4, 25 ) | Medium |  |
| Pointer | ANKC | ( 61, 69 ) | EoDB | ( 20.0, 34.0 ) | Large |  |
| Polish Lowland Sheepdog | ANKC | ( 42, 50 ) | EoDB | ( 13.6, 15.9 ) | Medium |  |
| Pomeranian | ANKC | ( 20, 28 ) | ANKC | ( 1.8, 2.0 ) | Small | 75.90 ( ±3.53 ) |
| Poodle (Miniature) | EoDB | ( 25, 38 ) | EoDB | ( 5.4, 8.2 ) | Small |  |
| Poodle (Standard) | EoDB | ( 38, NA ) | EoDB | ( 20.4, 29.5 ) | Medium |  |
| Poodle (Toy) | EoDB | ( NA, 25 ) | EoDB | ( 1.8, 3.6 ) | Small |  |
| Portugese Water Dog | ANKC | ( 43, 57 ) | ANKC | ( 20.0, 30.0 ) | Large |  |
| Portuguese Podengo (Large) | ANKC | ( 55, 70 ) | ANKC | ( 16.0, 20.0 ) | Medium |  |
| Portuguese Podengo (Medium) | ANKC | ( 40, 54 ) | ANKC | ( 4.0, 6.0 ) | Small |  |
| Portuguese Podengo (Small) | ANKC | ( 20, 30 ) | ANKC | ( 16.0, 25.0 ) | Medium |  |
| Pug | EoDB | ( 25, 28 ) | ANKC | ( 6.3, 8.1 ) | Small | 98.55 ( ±6.74 ) |
| Puli | ANKC | ( 37, 44 ) | ANKC | ( 10.0, 15.0 ) | Medium |  |
| Pumi | ANKC | ( 38, 47 ) | ANKC | ( 8.0, 15.0 ) | Medium |  |
| Pyrenean Mastiff | ANKC | ( 72, 77 ) |  |  |  |  |
| Pyrenean Mountain Dog | ANKC | ( 65, 70 ) | ANKC | ( 40.0, 50.0 ) | Giant |  |
| Pyrenean Sheepdog Longhaired | ANKC | ( 40, 48 ) |  |  |  |  |
| Rhodesian Ridgeback | ANKC | ( 61, 69 ) | EoDB | ( 31.8, 38.6 ) | Large | 50.45 ( ±3.10 ) |
| Rottweiler | ANKC | ( 56, 68 ) | EoDB | ( 36.3, 61.2 ) | Giant | 63.55 ( ±2.95 ) |
| Russian Black Terrier | ANKC | ( 68, 76 ) | ANKC | ( 45.0, 60.0 ) | Giant |  |
| Russian Toy | ANKC | ( 20, 28 ) | ANKC | ( NA, 3.0 ) | Small |  |
| Saluki | ANKC | ( 58, 71 ) | EoDB | ( 15.9, 29.5 ) | Medium | 45.85 ( ±8.77 ) |
| Samoyed | ANKC | ( 46, 56 ) | EoDB | ( 15.9, 27.2 ) | Medium | 60.40 ( ±8.27 ) |
| Schipperke | EoDB | ( 25, 33 ) | ANKC | ( 5.5, 7.5 ) | Small |  |
| Schnauzer | EoDB | ( 44, 50 ) | EoDB | ( 18.1, 20.4 ) | Medium | 53.65 ( ±2.63 ) |
| Schnauzer (Giant) | ANKC | ( 60, 70 ) | EoDB | ( 29.5, 40.8 ) | Large |  |
| Schnauzer (Miniature) | EoDB | ( 30, 36 ) | EoDB | ( 5.9, 6.8 ) | Small | 53.40 ( ±2.44 ) |
| Scottish Terrier | ANKC | ( 25, 28 ) | ANKC | ( 8.5, 10.5 ) | Small | 45.85 ( ±9.14 ) |
| Sealyham Terrier | ANKC | ( 31, 31 ) | EoDB | ( 8.2, 10.9 ) | Small |  |
| Shar Pei | ANKC | ( 46, 51 ) | EoDB | ( 20.4, 27.2 ) | Medium |  |
| Shetland Sheepdog | EoDB | ( 33, 41 ) | EoDB | ( 9.1, 9.1 ) | Small | 50.45 ( ±4.18 ) |
| Shiba Inu | EoDB | ( 34, 42 ) | EoDB | ( 7.7, 10.4 ) | Small |  |
| Shih Tzu | ANKC | ( NA, 27 ) | ANKC | ( 4.5, 7.5 ) | Small | 79.45 ( ±7.00 ) |
| Siberian Husky | ANKC | ( 51, 60 ) | ANKC | ( 16.0, 27.0 ) | Medium | 54.90 ( ±3.71 ) |
| Skye Terrier | ANKC | ( 25, 26 ) | EoDB | ( 15.9, 18.1 ) | Medium |  |
| Sloughi | ANKC | ( 61, 72 ) |  |  |  |  |
| Soft Coated Wheaten Terrier | ANKC | ( 46, 49 ) | ANKC | ( 16.0, 20.5 ) | Medium |  |
| Spanish Mastiff | ANKC | ( 75, 80 ) |  |  |  |  |
| St Bernard | ANKC | ( 65, 90 ) | EoDB | ( 54.4, 90.7 ) | Giant |  |
| Staffordshire Bull Terrier | ANKC | ( 36, 41 ) | ANKC | ( 11.0, 17.0 ) | Medium | 76.15 ( ±6.32 ) |
| Sussex Spaniel | ANKC | ( 38, 41 ) | EoDB | ( 15.9, 20.4 ) | Medium |  |
| Swedish Lapphund | ANKC | ( 40, 51 ) |  |  |  |  |
| Swedish Vallhund | ANKC | ( 31, 35 ) | ANKC | ( 11.5, 16.0 ) | Medium |  |
| Tatra Shepherd Dog | ANKC | ( 60, 70 ) |  |  |  |  |
| Tenterfield Terrier | ANKC | ( 26, 31 ) |  |  |  | 59.75 ( ±6.85 ) |
| Tibetan Mastiff | ANKC | ( 61, 66 ) | EoDB | ( 36.3, 68.0 ) | Giant |  |
| Tibetan Spaniel | ANKC | ( NA, 25 ) | ANKC | ( 4.1, 6.8 ) | Small | 83.85 ( ±5.81 ) |
| Tibetan Terrier | ANKC | ( 36, 41 ) | EoDB | ( 8.2, 13.6 ) | Medium | 58.35 ( ±3.38 ) |
| Weimaraner | ANKC | ( 57, 70 ) | ANKC | ( 25.0, 40.0 ) | Large | 49.90 ( ±8.22 ) |
| Weimaraner (Longhair) | ANKC | ( 57, 70 ) | ANKC | ( 25.0, 40.0 ) | Large |  |
| Welsh Corgi (Cardigan) | EoDB | ( 27, 32 ) | EoDB | ( 11.3, 17.2 ) | Medium | 62.30 ( ±5.32 ) |
| Welsh Corgi (Pembroke) | ANKC | ( 25, 30 ) | ANKC | ( 9.0, 12.0 ) | Medium |  |
| Welsh Springer Spaniel | EoDB | ( 43, 48 ) | EoDB | ( 11.3, 12.3 ) | Medium |  |
| Welsh Terrier | ANKC | ( NA, 39 ) | ANKC | ( 9.0, 9.5 ) | Small |  |
| West Highland White Terrier | ANKC | ( 28, 28 ) | EoDB | ( 6.8, 9.5 ) | Small | 64.40 ( ±6.45 ) |
| Whippet | ANKC | ( 44, 51 ) | EoDB | ( 9.1, 18.1 ) | Medium | 51.40 ( ±5.89 ) |
| White Swiss Shepherd Dog | ANKC | ( 55, 66 ) |  |  |  |  |
| Yorkshire Terrier | EoDB | ( 20, 23 ) | ANKC | ( NA, 3.2 ) | Small |  |

**Reference**

1.	Australian National Kennel Council: **Breed standard.** <http://ankc.org.au/Breed/Index>. Access 28 Jan 2015.

2.	Coile DC: **Encyclopedia of dog breeds**. Hauppauge, NY: Barron's Educational Series; 2005.

3.	Carrasco JJ, Georgevsky D, Valenzuela M, McGreevy PD: **A pilot study of sexual dimorphism in the head morphology of domestic dogs**. *J Vet Behav* 2014, **9**(1):43-46.
